# Publisher Correction: Loss of POMC-mediated antinociception contributes to painful diabetic neuropathy

**DOI:** 10.1038/s41467-021-21406-x

**Published:** 2021-02-08

**Authors:** Divija Deshpande, Nitin Agarwal, Thomas Fleming, Claire Gaveriaux-Ruff, Christoph S. N. Klose, Anke Tappe-Theodor, Rohini Kuner, Peter Nawroth

**Affiliations:** 1 Department of Medicine I and Clinical Chemistry, University Hospital of Heidelberg, INF 410, Heidelberg, Germany; 2 Institute of Pharmacology, Heidelberg University, INF 366, Heidelberg, 69120 Germany; 3 Department of Microbiology, Infectious Diseases and Immunology, Charité-Universitätsmedizin Berlin, Hindenburgdamm 30, 12203 Berlin, Germany; 4 German Center for Diabetes Research (DZD), Neuherberg, Germany; 5 Institut de Génétique et de Biologie Moléculaire et Cellulaire, Department of Translational Medicine and Neurogenetics, Illkirch, France; 6 Université de Strasbourg, Illkirch, France; 7 Centre National de la Recherche Scientifique, UMR7104 Illkirch, France; 8 Institut National de la Santé et de la Recherche Médicale, U1258 Illkirch, France; 9 Ecole Supérieure de Biotechnologie de Strasbourg, Illkirch, France; 10 Joint Heidelberg-IDC Translational Diabetes Program, Helmholtz Zentrum, 85764 Neuherberg, Germany

**Keywords:** Cell biology, Molecular biology, Neuroscience, Diseases, Endocrinology

Correction to: *Nature Communications* 10.1038/s41467-020-20677-0, published online 18 January 2021.

The original version of the Peer Review File associated with this Article was updated shortly after publication to redact unpublished data.

