# Supplementary material for: Loss of POMC-mediated antinociception contributes to painful diabetic neuropathy
Source: Nat Commun. 2021 Jan 18;12:426. doi: 10.1038/s41467-020-20677-0 (PMC7814083; doi:10.1038/s41467-020-20677-0)
Supplement: Supplementary file 1 — Supplementary Information [file 41467_2020_20677_MOESM1_ESM.pdf]

SUPPLEMENTARY FIGURES

Table T1

Name	Sequence	Application
18srRNA_forward	GTA ACC CGT TGA ACC CCA TT	qPCR
18srRNA_reverse	CCA TCC AAT CGG TAG TAG CG	qPCR
POMC_forward	ATGCCGAGATTCTGCTACAGT	qPCR
POMC_reverse	TCCAGCGAGAGGTCGAGTTT	qPCR
Blocking peptide for goat anti-mouse POMC antibody	NAIIKNAYKKGE	IF, IB
Blocking peptide for rabbit anti-mouse POMC antibody	FPGNGDEQPLTENPRK	IF
MOR_forward	TCCGACTCATGTTGAAAAACCC	qPCR
MOR_reverse	CCTTCCCCGGATTCCTGTCT	qPCR
POMC promoter_forward	AGTTCTTCCTAACCACCAGCGCC	ChIP-qPCR
POMC promoter_reverse	TATACTTGCAGGGTTGGGTGGGTG	ChIP-qPCR
GAPDH_forward	CAGCCGGAGTTCTTAACCAG	ChIP-qPCR
GAPDH_reverse	CTGCCAATCCTGATGGACTAA	ChIP-qPCR
NF-kB consensus	E329a (Promega)	EMSA
POMC promoter_oligo	ATGGCTTGCATCCGGGCTTGCAAACTCGCCTCTCGACGCTGCAGATGGCTTGCATC CGG GCTTGCAAACTCGACC	Site-directed mutagenesis
POMC_forward + SbfI	ATATACCTGCAGGATGCCGAGATTCTGCTACAGTCGCTCAGGGGCC	Cloning of POMC-GFP in pAM vector
MOR_forward + SbfI	ATATACCTGCAGGATGGACAGCAGCGCCGGCCCAGGGAA	Cloning of MOR-GFP in pAM vector
GFP_reverse + EcoRI	ACGTGGAATTCCTTGTACAGCTCGTCCATGCCGAGAGTGATCCCG	Cloning of POMC and MOR GFP constructs
2A viral peptide	GGAAGCGGAGAGGGCAGAGGAAGTCTGCTAACATGCGGTGACGTCGAGGAGAAT CCTGGACCT	Cloning of bicistronic POMC-MOR construct

Table T1: Oligonucleotide and peptide sequences
Abbreviations: IF= immunoflourescence, IB= immunoblotting

Figure S1

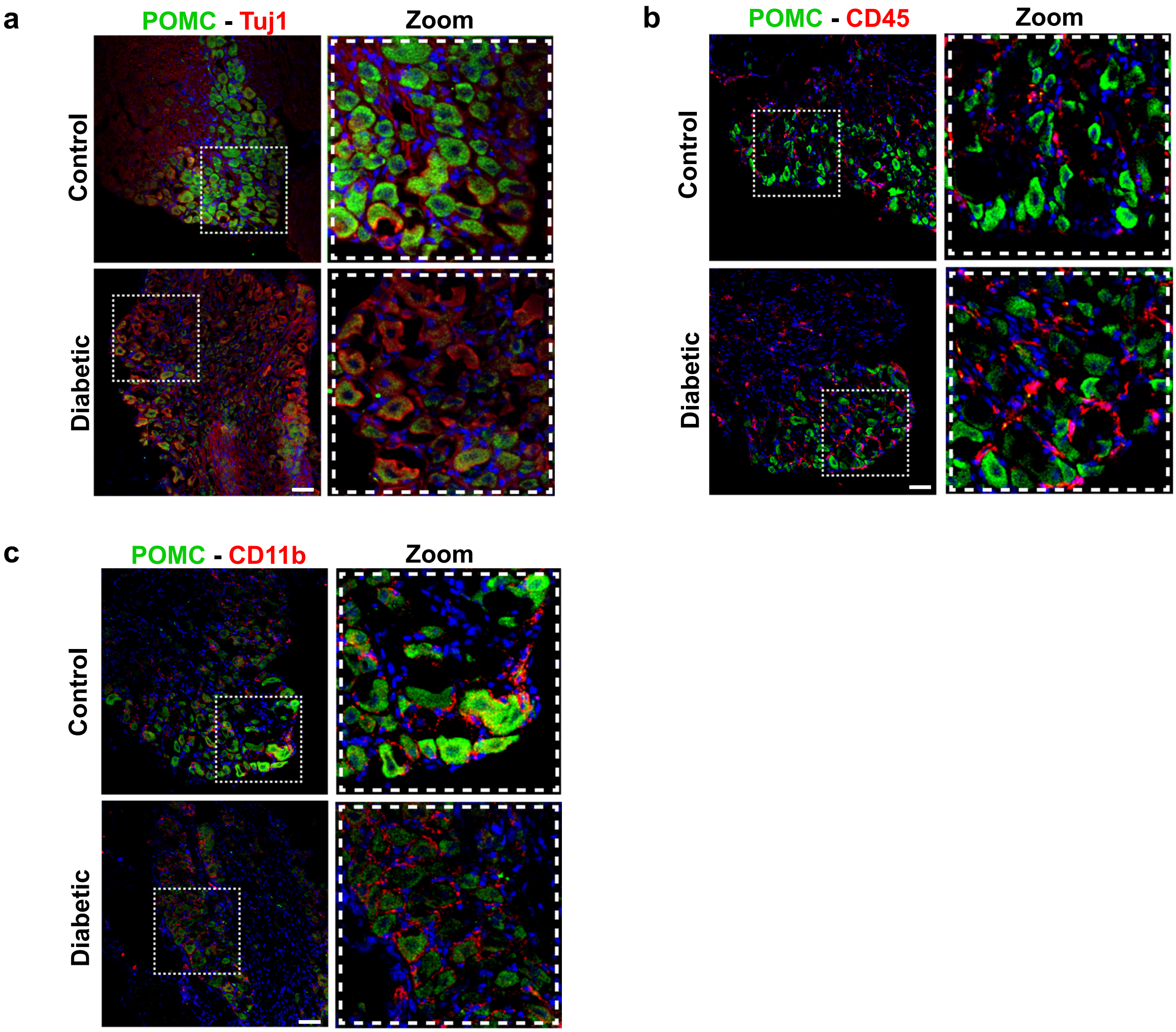

Fig. S1: POMC is expressed by neuronal cells in the DRG (related to Main Figure 1 and 2).
Co-staining of lumbar DRG of control and diabetic mice with cell specific markers for neurons (Tuj-1), immune cells (CD45) or glial cells (CD11b) along with Rb anti-POMC antibody, show that the neurons are the predominant cell type in the PNS to express POMC (n=6 mice/group, 5-10 DRG sections from each mouse). In panels (a), (b) and (c) areas are magnified to depict the colocalization between POMC-specific immunoreactivity and respective cell markers in control and diabetic lumbar DRG. Scale=50 μm.

Figure S2

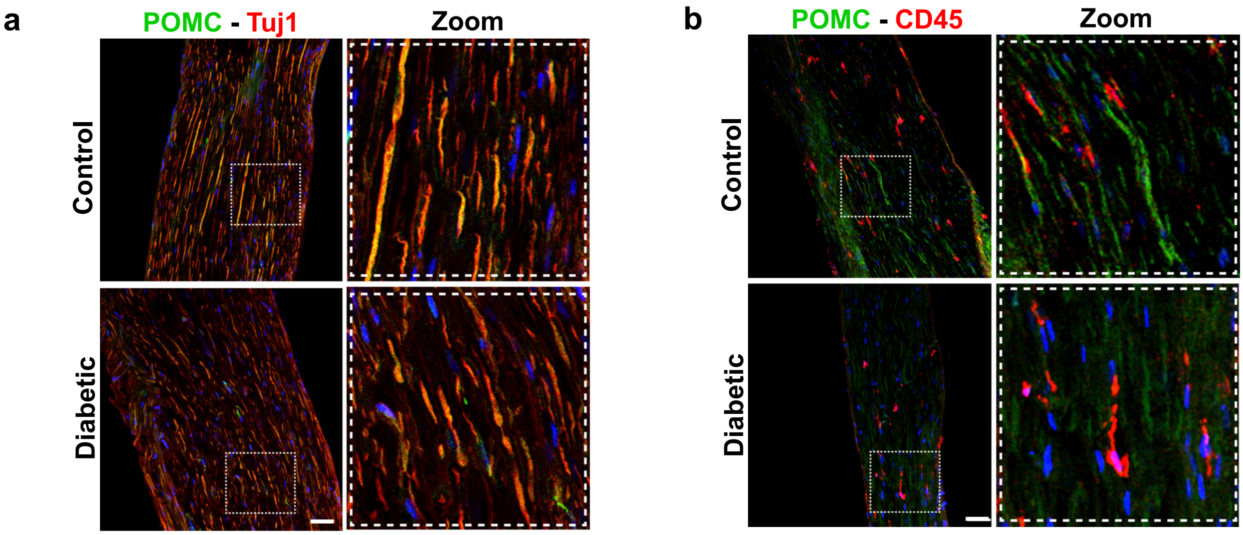

Fig. S2: POMC signal is in the axons of sciatic nerves (related to Main Figure 1 and 2).
Co-staining of sciatic nerves of control and diabetic mice with cell specific markers for neurons (Tuj-1), immune cells (CD45) along with anti-POMC Rb antibody, show that the POMC signal is predominantly located in the axons of the sciatic nerves (n=6 mice/group, 6-10 DRG sections from each mouse). In panels (a) and (b) areas are magnified depict the colocalization between POMC-specific immunoreactivity and respective cell markers in control and diabetic sciatic nerves. Scale=50 μm.

Table T2

**Age**	78.6 +/- 4 years
**Gender**	male (n=3) ; female (n=3)
**Diabetes (type-1 and type-2)**	no
**Reason for Amputation**	cancer/cachexia
**Nephropathy/CKD**	absent
**Neuropathy**	absent
**Retinopathy**	absent
**CVD**	present in 50% cases
**Hypertension**	present in 50% cases

Table T2: Characteristics of healthy human subjects analyzed for POMC expression in lumbar DRG (related to Main Figure 1)

Abbreviations: CVD= cardio-vascular disease CKD= Chronic Kidney Disease

Figure S3

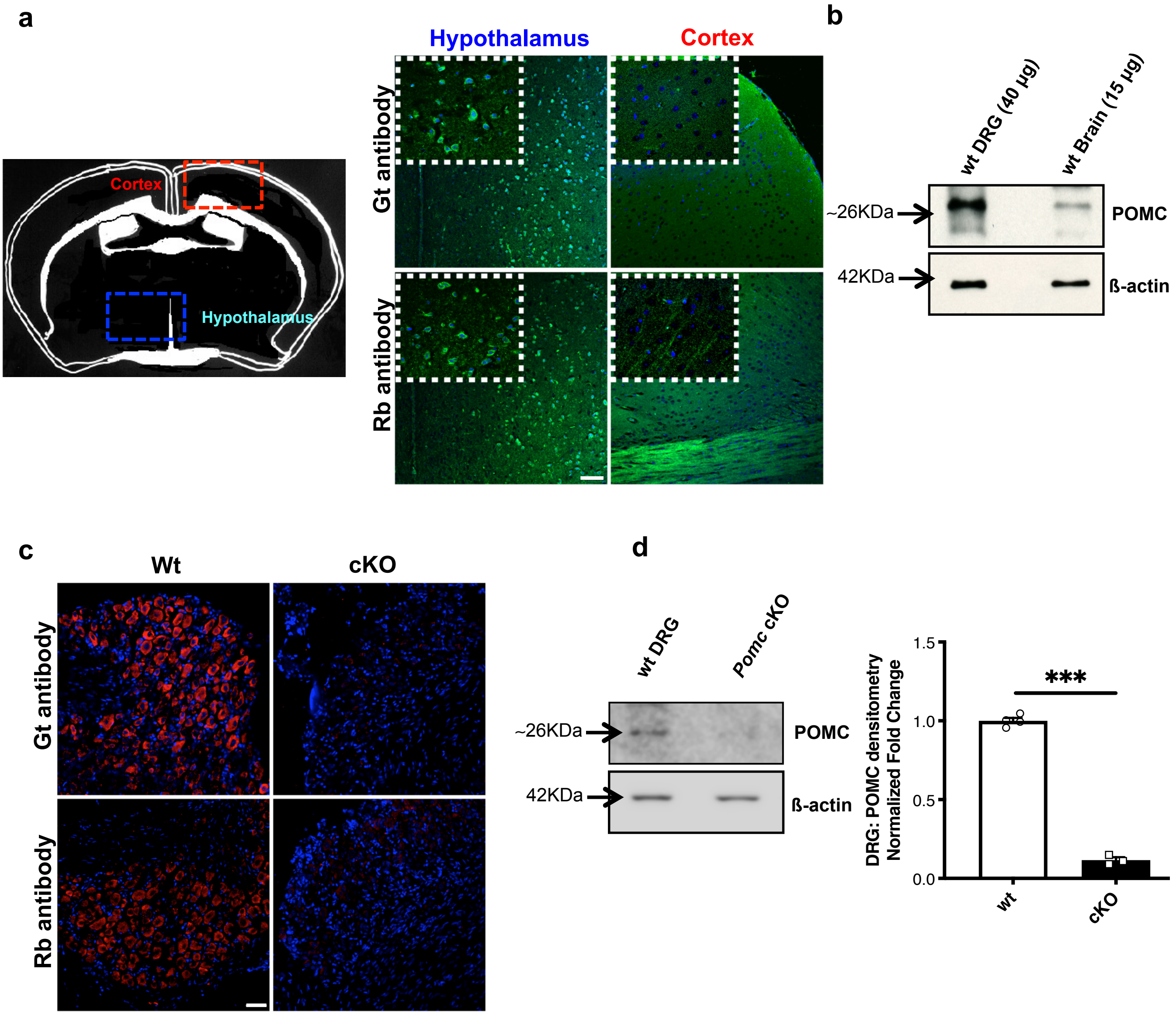

Fig. S3: POMC antibody specificity (related to Main Figure 1, 2).
Specificity of the anti-POMC antibodies shown in **a, b** immunofluorescence and **c, d** immunoblot using mouse brain as a positive control and *Pomc* conditional knockout (cKO; lacking *Pomc* expression in all neurons expressing Snap25 protein generated using cre-lox system) as negative control. **a** Naïve control mouse brain sections immunostained using either anti-POMC **a** Goat (Gt; #PA518368) antibody or Rabbit (Rb; #23499) antibody detect specific signal in hypothalamus region known to express POMC, whereas no signal in neuronal somata of the cortex region. Inset shows a magnified image of neuronal soma (n=3/group) **b** Immunoblot showing the detection of the same ~26 KDa POMC band in the total protein lysates of mouse brain and lumbar DRG using anti-POMC Gt antibody (repeated thrice). **c** Absence of POMC immunostaining in the cKO DRG sections compared to the wild-type (Wt) DRGs further show specificity of the anti-POMC Gt and Rb antibodies (n=4/group). **d** The detection of ~26 KDa POMC band in the Wt DRG lysates and the loss of the same band in cKO DRG lysates confirm the specificity of anti-POMC Gt antibody (wt, n=4 and cko, n=3; two tailed t-test;***p<0.0001) and indicate that the specific bands with higher molecular weight bands observed are probably the oligomers of POMC peptide. Data represents mean ± SEM, 95% C.I. Circles represent individual data points. Scale=50 µm. Source data are provided as a Source Data file.

Figure S4

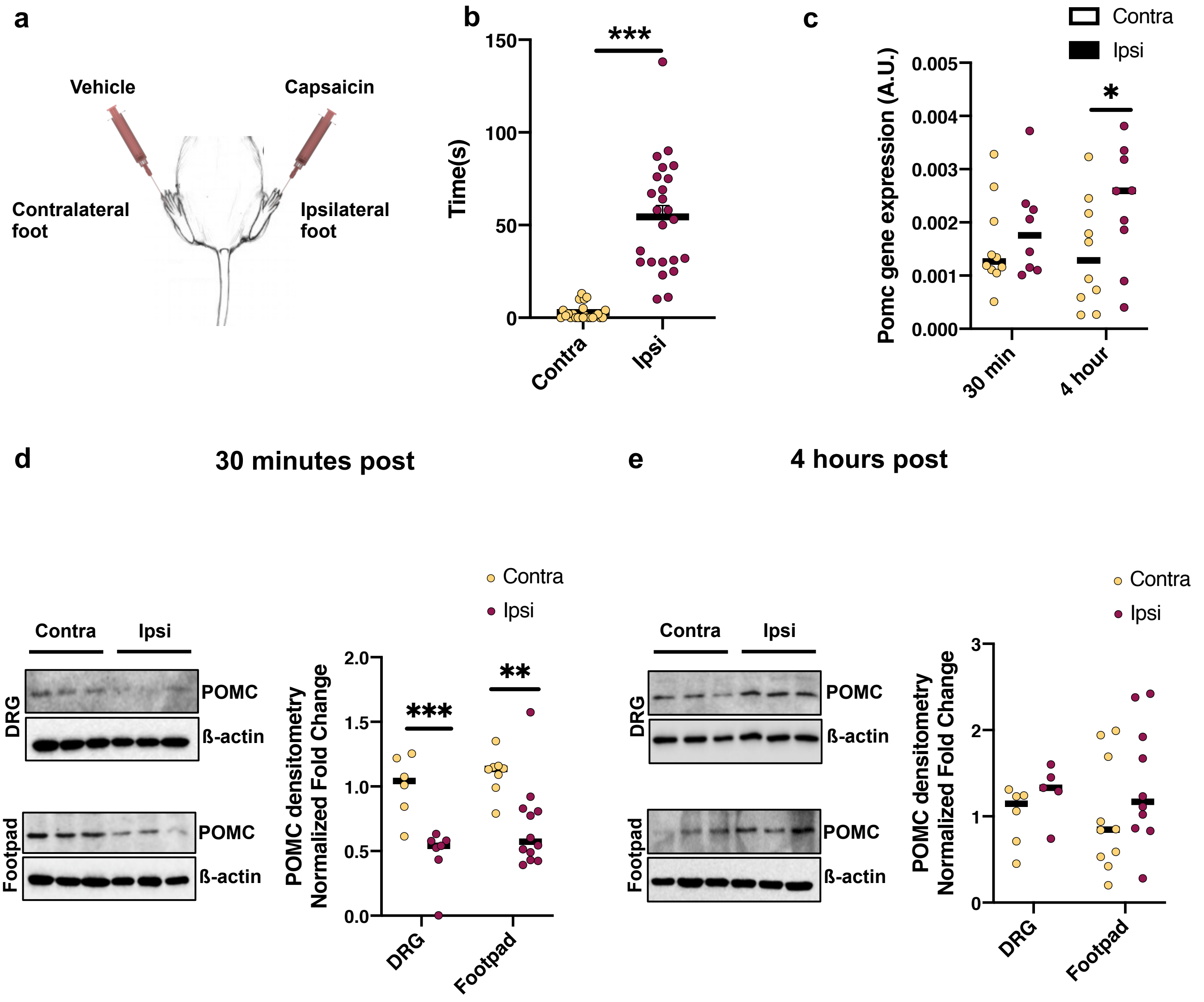

Fig. S4: POMC in PNS participates to resolve capsaicin-induced acute pain (related to Main Figure 1).
a Schematic showing injection of capsaicin (0.3%) in one foot (ipsilateral) and vehicle in the other foot (contralateral). **b** The mice spent significant time showing nocifensive behavior only for the capsaicin-injected ipsilateral foot during the 10 minutes observation period, after which no such behavior is observed. (n=24 for contra and ipsi readings; two tailed t-test; ***p<0.0001) **c** Pomc gene expression quantified in the lumbar DRGs shows a significant increase 4 hours post capsaicin injection (DRG, n=10 for ipsi and n=8 for contra; footpads, n=10 for ipsi and n=9 for contra; two tailed t-test; **p=0.004, ***p=0.001). **d** A significant loss of POMC protein is observed in the lumbar DRGs and footpads 30 minutes post injection indicating release of the neuropeptide (DRG, n=6 for ipsi and n=7 for contra; footpads, n=8 for ipsi and n=12 for contra; two tailed t-test; **p=0.004, ***p=0.001). **e** The POMC protein levels are restored at 4 hours post injection in the lumbar DRGs and the footpads (DRG, n=6 for ipsi and n=5 for contra; footpads, n=10 for ipsi and n=10 for contra; two tailed t-test). For all panels, data shown as dot plot with median, 95% C.I.; circles represent individual data points; orange circle: contra and maroon circle: ipsi data. Pomc band corresponds to ~26KDa and actin to ~42KDa. Source data are provided as a Source Data file.

Table T3

	a	b	c	d	e	f
♀	**Control**	**Diabetic**	**D + GFP**	**D + MOR**	**D+POMC**	**D + POMC-MOR**
n	20	20	10	10	10	10
**Blood glucose** (mg/dL)	116.7 ± 2.2	320.9 ± 19.06 ***	324.2 ± 29.50 ***	317.9 ± 23.92 ***	376.4 ± 15.62 ***	324.5 ± 8.81 ***
**Body weight** (g)	25.72 ± 0.28	19.34 ± 0.61 ***	20.4 ± 0.58 ***	21.2 ± 0.42 ***	21.8 ± 0.23 ***	21.4 ± 0.36 ***
**Hba1c** (%)	2.98 ± 0.09	10.2 ± 0.5 ***	5.8 ± 0.39 ***	5.07 ± 0.26 **	5.8 ± 0.44 ***	5.31 ± 0.22 ***

Table T3: Diabetic parameters of mice
a - f Diabetic parameters measured in female (♀) mice. **b** 12 weeks after STZ induction, the mice display significantly increased blood glucose levels and HbA1c content, while body weight was decreased as compared with the **a** age-matched naïve controls. **c-f** Diabetic mice injected with AAV-constructs overexpressing the genes GFP, MOR, POMC or POMC-MOR also have elevated blood glucose levels and HBa1c, while body weight was decreased compared to the naïve control mice. In panels (a-f), data represents mean ± SEM with 95% C.I., all values compared to the corresponding values of control mice using one-way ANOVA followed by Dunnett's post-hoc test; **p=0.001, ***p<0.0001. Source data are provided as a Source Data file.

Table T4

	a	b	c	d
♂	**Control**	**Diabetic**	**D + GFP**	**D + POMC-MOR**
n	10	11	8	8
**Blood glucose** (mg/dL)	96.5 ± 3.75	425.36 ± 25.3 ***	493.77 ± 21.56 ***	456.66 ± 38.55 ***
**Body weight** (g)	27.74 ± 0.61	24.13 ± 0.54 ***	23.93 ± 0.42 ***	25.45 ± 0.47 **
**Hba1c** (%)	4.83 ± 0.14	12.37 ± 0.29 ***	11.56 ± 0.39 ***	10.27 ± 0.45 ***

Table T4: Diabetic parameters of mice
a - d Diabetic parameters measured in male (♂) mice. **b** 6 weeks after STZ induction, the mice display significantly increased blood glucose levels and HbA1c content, while body weight was decreased as compared with the **a** age-matched naïve controls. **c, d** Diabetic mice injected with AAV-constructs overexpressing the genes GFP, MOR, POMC or POMC-MOR also have elevated blood glucose levels and HBa1c, while body weight was decreased compared to the naïve control mice. In panels (a-d), data represents mean ± SEM with 95% C.I., all values compared to the corresponding values of control mice using one-way ANOVA followed by Dunnett's post-hoc test; **p=0.006, ***p<0.0001. Source data are provided as a Source Data file.

Figure S5

a

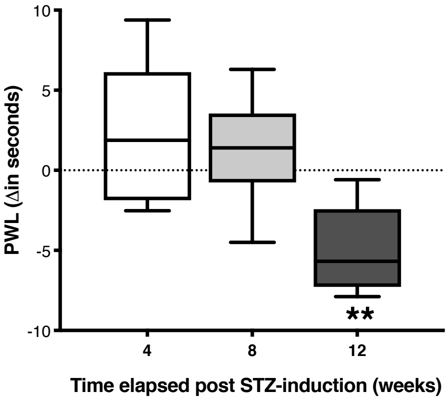

b

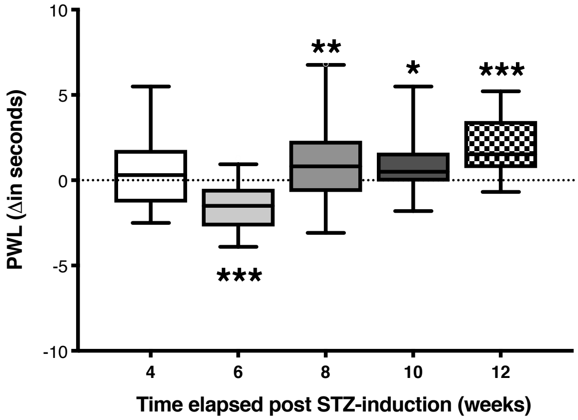

Fig. S5: Thermal hyperalgesia profile of female and male diabetic mice after STZ induction (related to Main Figure 1 and 2).
a Thermal hyperalgesia measured in female mice using Hotplate test (50°C) after 4 (n=9), 8 (n=9), and 12 (n=9) weeks post STZ induction (two tailed t-test; *p=0.005). The diabetic female mice recover from the initial hyperalgesia until 8 weeks and display a significant hyperalgesia again at 12 weeks post induction. **b** Thermal hyperalgesia measured in male mice using Hargreaves test (50°C) after 4 (n=8), 6 (n=10), 8 (n=10), 10 (n=8) and 12 (n=8) weeks post STZ induction (two tailed t-test; *p=0,03, **p=0.001,***p<0.001). The diabetic male mice display a significant hyperalgesic phase at 6 weeks and then a tendency towards hypoalgesia until 12 weeks post STZ induction. In both panels, the boxplots corresponds to median, 25th and 75th percentiles, whiskers correspond to minimum and maximum values, 95% C.I. For each time point, values normalized with those of age-matched naïve control mice(Δ) and statistical comparison made for diabetic and the corresponding non-diabetic cohort at a particular time point. PWL=Paw Withdrawal Latency. Source data are provided as a Source Data file.

Figure S6

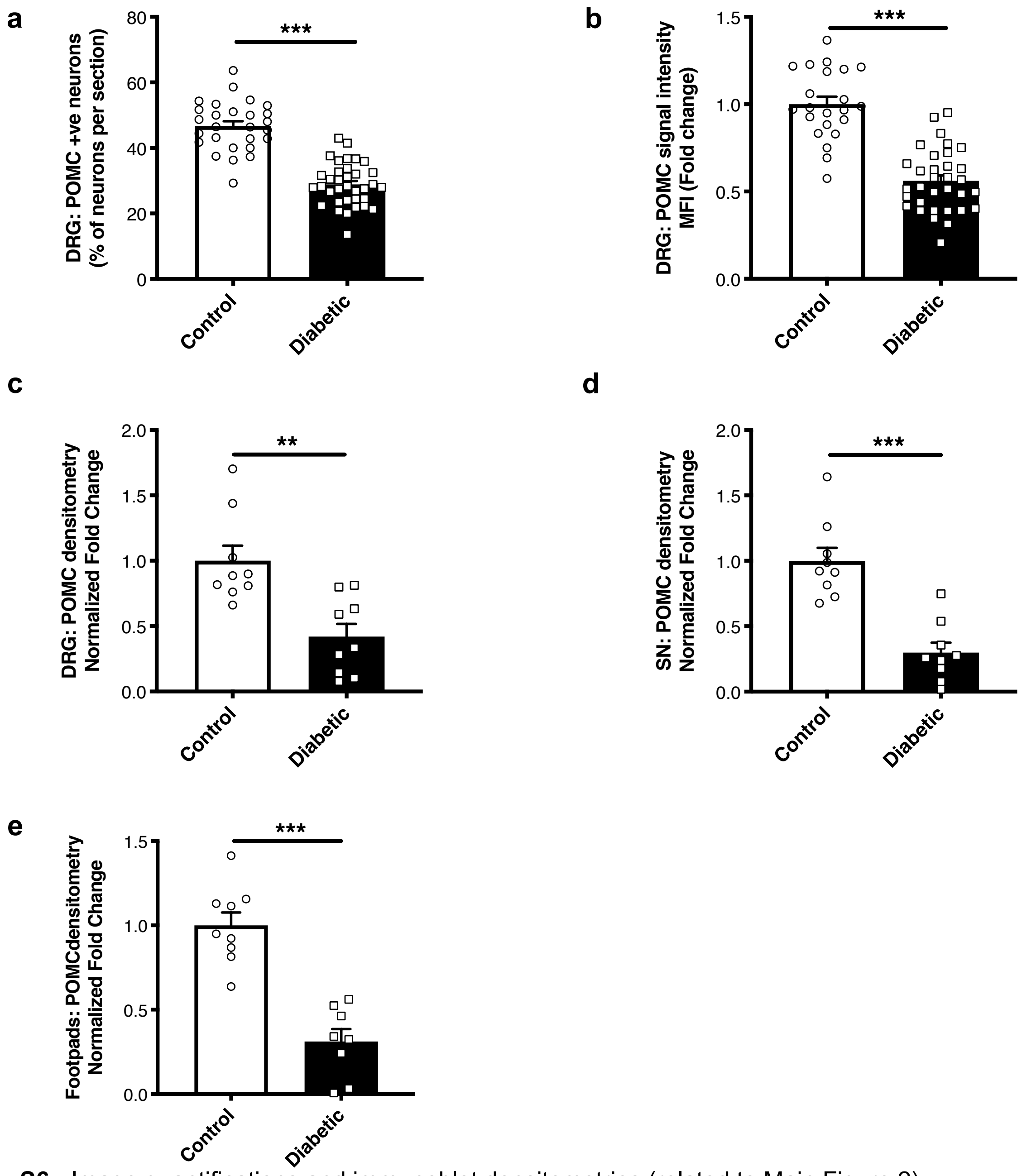

Fig. S6: Image quantifications and immunoblot densitometries (related to Main Figure 2).
Image quantification performed on DRG sections co-stained using anti-POMC Rb antibody and anti-Tuj-1 antibody demonstrate **a** decreased number of Tuj-1+ neurons expressing POMC in the DRG of diabetic mice and also **b** significantly lower Mean fluorescence intensity (MFI) of POMC immunoreactivity compared to the DRG of control mice (control, n=5 and diabetic, n=5; 5-9 sections for each mouse anaylzed for panels (a) and (b). Comparison of densitometric data from ~26 KDa POMC band detected using anti-POMC Gt antibody (normalized with actin band density) shows a significant decrease in POMC protein level in the total lysates of **c** DRG (control, n=9 and diabetic, n=9; **p=0.0014) **d** sciatic nerves (SN; control, n=9 and diabetic, n=9;***p<0.0001) and **e** footpads (control, n=9 and diabetic, n=8;***p<0.0001) of diabetic mice compared to controls. For all data represents at mean ± SEM; two tailed t-test with 95% C.I. Circles and squares represent individual data points. Source data are provided as a Source Data file.

Figure S7

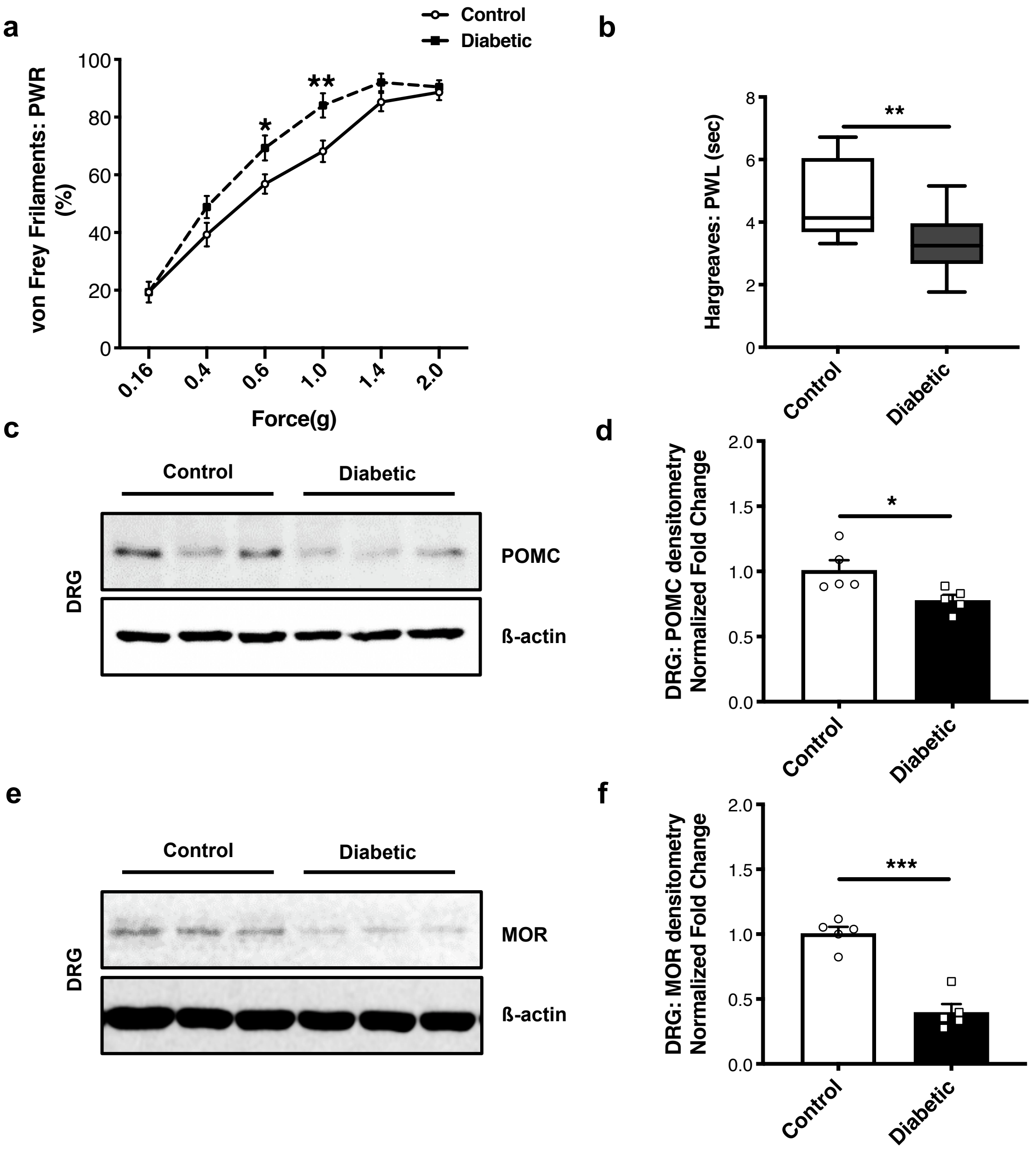

Fig. S7: POMC and MOR are downregulated in diabetic mice (related to Main Figure 2, 3).
6 weeks STZ-treated diabetic mice and age-matched controls (STZ-untreated) measured for **a** mechanical hyperalgesia (control denoted by circles, n=11 and diabetic denoted by squares, n=11; two-way ANOVA followed by Sidak's post-hoc test; **p=0.007). PWR=Paw Withdrawal Responses and **b** thermal hyperalgesia (control, n=11 and diabetic, n=11; two tailed t-test;**p=0.003). PWL=Paw Withdrawal Latency. Total protein lysates from DRG of 6-7 weeks STZ-treated male mice compared with age-matched controls for **c, d** POMC and **e, f** MOR protein levels using immunoblotting. **c** Representative blot showing comparison of POMC protein level (using Gt antibody) and the **d** densitometric data from ~26 KDa POMC band (normalized with actin band density) shows a significant decrease in POMC protein level in the DRG of diabetic male mice. **e** Representative blot showing comparison of MOR protein level and the **f** densitometric data from ~55 KDa MOR band (normalized with actin band density) shows a significant decrease in MOR protein level as well. For panels (b) the boxplot corresponds to median, 25th and 75th percentiles, whiskers correspond to minimum and maximum values, 95% C.I. For panels (d) and (f) (control, n=5 and diabetic, n=5) data represents mean ± SEM, 95% C.I. *p<0.027, ***p<0.0001; two tailed t-test. Circles represent individual data points. For all panels, male mice data is shown. Source data are provided as a Source Data file.

Figure S8

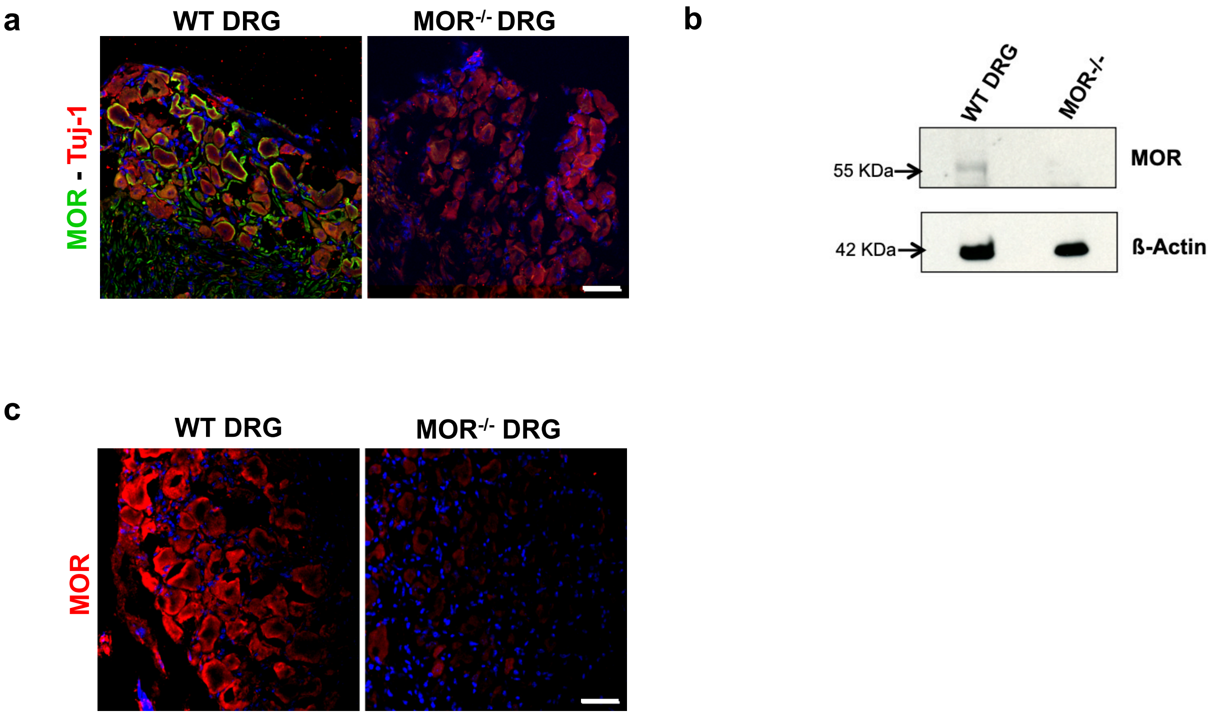

Fig. S8: MOR antibody specificity (related to Main Figure 3 and 9).
Antibody specificity for the anti-MOR antibody (#RA10104 used in main Figures 3b, 3c and 5d) demonstrated in immunostaining **a** and immunoblotting **b** using MOR$^{-/-}$ DRG and wild-type (WT) lumbar DRG (WT). **a** Co-immunostaining with #RA10104 anti-MOR antibody shows immunoreactivity in the Tuj-1+ neurons of WT DRG, and complete loss of immunoreactivity MOR$^{-/-}$ DRG (n=3/group). **b** Absence of ~55KDa band in MOR$^{-/-}$ DRG total protein lysate, which is detectable in WT DRG total protein lysate (50µg/well) confirmed the specificity of this antibody. Co-incubation of the immunoblot with ß-actin antibody shows equal protein loads in both wells (n=3/group). **c** Immunoflourescence staining with anti-MOR antibody (#NB100-1618 used in main Figure 9a) shows immunoreactivity in the WT DRG and no immunoreactivitiy in the MOR$^{-/-}$ DRG confirming the specificity of this antibody in immunoflourescence staining (n=3/group). Scale=50 µm. Source data are provided as a Source Data file.

Figure S9

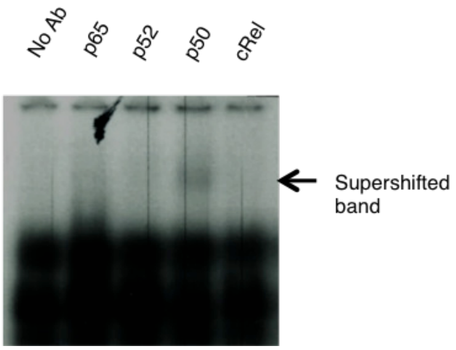

Fig. S9: NF-kB p50 subunit DNA binding is increased in HG exposed AtT-20 cells (related to Main Figure 4). NF-kB subunit activation measured in AtT-20 cell nuclear lysates using a supershift EMSA post exposure to high glucose for 12 hours. A supershift only in the NF-kB p50 subunit was observed.

Figure S10

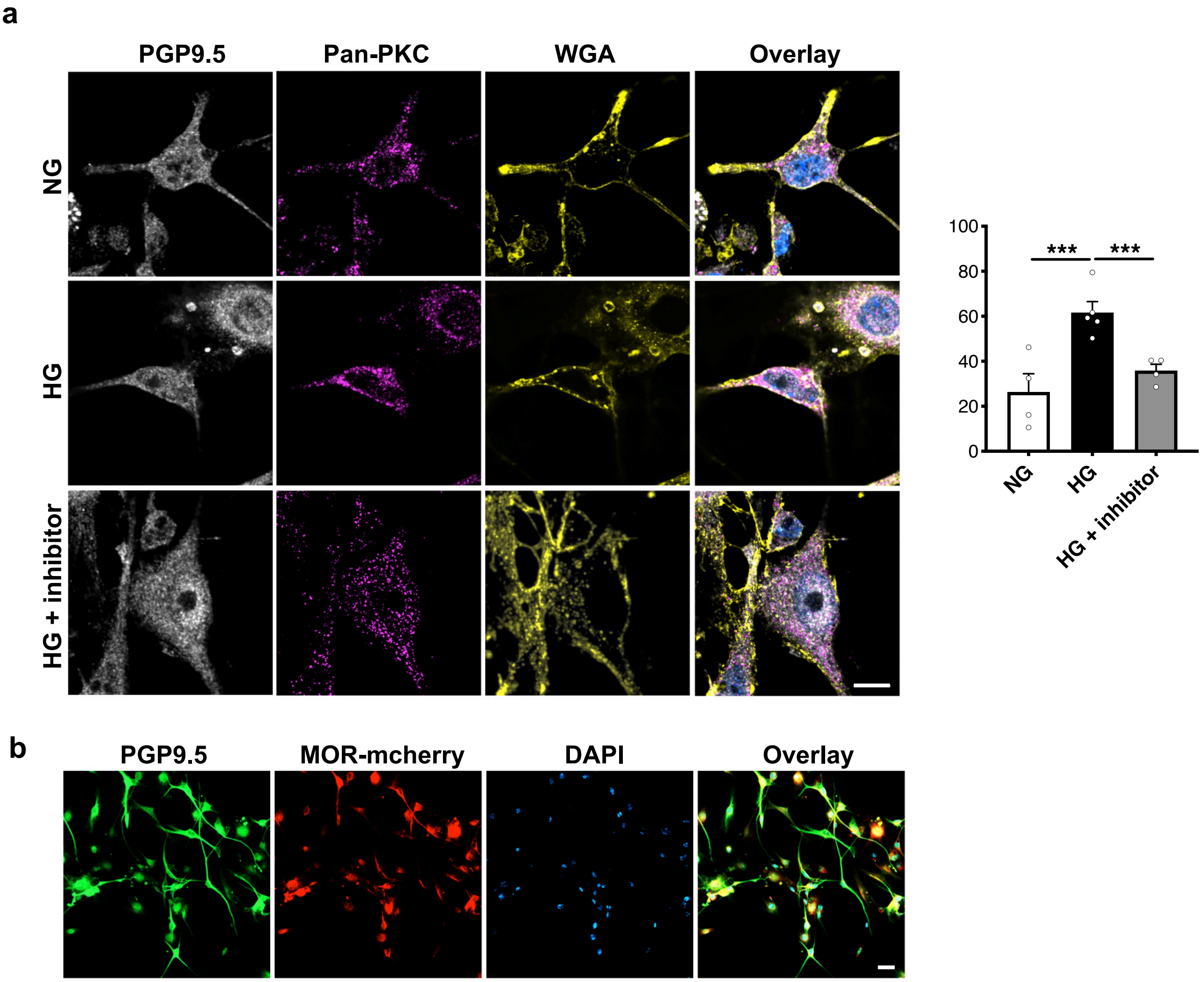

Fig. S10: Increased PKC activation in primary DRG neurons under hyperglycaemic conditions (related to Main Figure 5).

a Increased PKC activation is observed in cultured DRG cells exposed to high glucose (HG; 40 mM) as compared normal glucose exposure (NG; 17.5 mM) for 48 hours. PKC activation was determined by its subcellular localization. In inactive state, PKC is present within the neuronal cell body, whilst upon activation, the PKC signal is relocated to the cell membrane (denoted by Wheat Germ Agglutinin; WGA). In the presence of PKC inhibitor (Gö6983, 1 μM; 48h), PKC signal is re-localized within the neuronal cell body (NG, n=4; HG, n=5; HG + inhibitor, n=4). Data represents as mean + SEM; ***$p<0.0001$; with 95% C.I. one-way ANOVA followed by Dunnett's post-hoc test. Circles represent individual data points. **b** Co-immunostaining of the MOR-mCherry transfected DRG cells with anti-PGP9.5 (pan-neuronal marker) shows the DRG culture consists mainly of neuronal cells and that these neuronal cells were transfected with the construct (n=5). Scale=10 μm. Source data are provided as a Source Data file.

Table T5

	Naïve control mice					
**Force**	**0.16g**	**0.4g**	**0.6g**	**1.0g**	**1.4g**	**2.0g**
**GFP**	0,4114	**0,0012	***0,0001	***0,0001	**0,0057	*0,0554
**MOR**	0,5600	**0,0010	***0,0001	***0,0001	**0,0057	0,1528
**POMC**	0,0554	***0,0001	***0,0001	***0,0001	***0,0006	*0,0312
**POMC-MOR**	*0,0200	***0,0001	***0,0001	***0,0001	***0,0001	0,2144

Table T5: Significance values of contralateral hindpaw before PKC inhibitor injection (related to main Figure 6c).
Two-way ANOVA analysis followed by Dunnett's post-hoc test. 95%C.I. Source data are provided as a Source Data file.

Table T6

	GFP mice					
**Force**	**0.16g**	**0.4g**	**0.6g**	**1.0g**	**1.4g**	**2.0g**
**Naïve control**	0,2854	*0,0027	***0,0001	***0,0001	***0,0001	*0,0321
**MOR**	0,9997	0,9997	0,4949	0,9009	0,9996	0,9600
**POMC**	0,2164	*0,0120	***0,0001	***0,0001	**0,0015	*0,0106
**POMC-MOR**	0,5320	*0,0221	***0,0001	**0,0010	*0,0205	0,3709

Table T6: Significance values of ipsilateral hindpaw before PKC inhibitor injection (related to main Figure 6d).
Two-way ANOVA analysis followed by Dunnett's post-hoc test. 95%C.I. Source data are provided as a Source Data file.

Table T7

	Naïve control mice					
**Force**	**0.16g**	**0.4g**	**0.6g**	**1.0g**	**1.4g**	**2.0g**
**GFP**	0,0334	***0,0001	***0,0001	***0,0001	***0,0001	**0,0089
**MOR**	0,1179	***0,0001	***0,0001	***0,0001	***0,0001	*0,0061
**POMC**	0,2996	***0,0001	***0,0001	***0,0001	***0,0004	*0,0061
**POMC-MOR**	0,6057	***0,0001	***0,0001	***0,0001	***0,0001	**0,0089

Table T7: Significance values of contralateral hindpaw after PKC inhibitor injection (related to main Figure 6e).
Two-way ANOVA analysis followed by Dunnett's post-hoc test. 95%C.I. Source data are provided as a Source Data file.

Table T8

	GFP mice					
**Force**	**0.16g**	**0.4g**	**0.6g**	**1.0g**	**1.4g**	**2.0g**
**Naïve control**	0,3091	0,0883	0,7753	0,3323	0,8844	0,9999
**MOR**	0,8266	0,9457	0,9644	0,9996	0,6616	0,9997
**POMC**	*0,0286	***0,0005	***0,0004	***0,0001	*0,0130	0,9028
**POMC-MOR**	*0,0133	***0,0003	***0,0001	***0,0001	*0,0133	0,5715

Table T8: Significance values of ipsilateral hindpaw after PKC inhibitor injection (related to main Figure 6f).
Two-way ANOVA analysis followed by Dunnett's post-hoc test. 95%C.I. Source data are provided as a Source Data file.

Figure S11

a

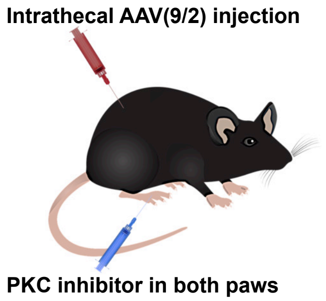

b

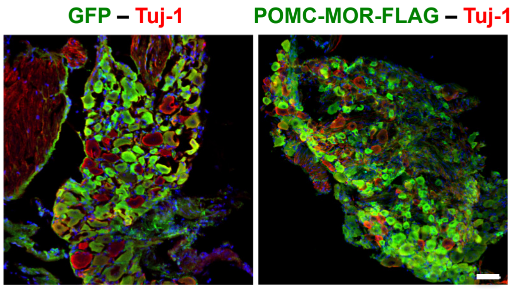

c

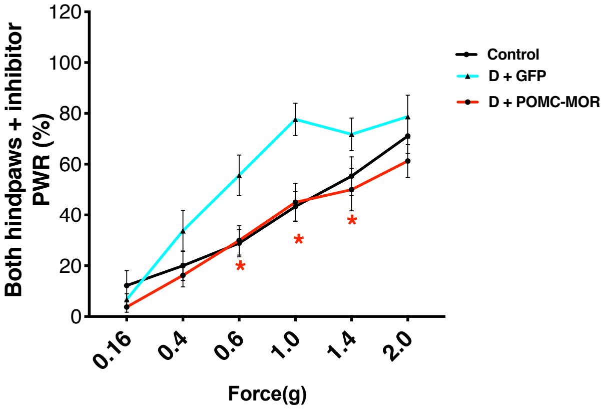

	GFP mice					
**Force**	**0.16g**	**0.4g**	**0.6g**	**1.0g**	**1.4g**	**2.0g**
**Naïve control**	0,7552	0,2258	**0,0052	***0,0003	0,1265	0,6084
**POMC-MOR**	0,9284	0,1116	**0,0100	***0,0009	*0,0354	0,1116

Fig. S11: Genetic reconstitution of POMC and MOR rescues mechanical hyperalgesia in diabetic mice (related to main Figure 6)
a Schematic representation of intrathecal injection in male diabetic mice with rAAV-9/2 constructs for overexpressing GFP or POMC-MOR. Readings taken after intraplantar injection of PKC inhibitor in both hindpaws (Gö6983, 20 µM, 45 minutes). Measurements were performed 6 weeks post STZ-induction. **b** Immunostaining of AAV-injected DRG with GFP or FLAG specific antibodies showing expression of the viral constructs (Naïve control, n=9; D+GFP, N=9 D+POMC-MOR, n=8). **c** Mechanical hypersensitivity measured in both hindpaws using von Frey filaments in naïve control (non-diabetic, non-AAV injected), and AAV-9/2 injected diabetic mice. (Naïve control, n=9; D+GFP, n=9 D+POMC-MOR, n=8). PWR= Paw Withdrawal responses. *(red star) indicates significant difference between D+POMC-MOR mice and D+GFP mice readings. Significance values listed in the adjoining Table. Data represents mean ± SEM with 95% C.I.; two-way ANOVA followed by Dunnett's post-hoc test. For all panels, male mice data is shown. Cyan line: D+GFP, red line: D+POMC-MOR. Scale=50 µm. Source data are provided as a Source Data file.

Table T9

	GFP mice				
**Force**	**10**	**15**	**20**	**25**	**30**
**Naïve control**	0,3739	0,4735	0,3328	0,4969	0,5746
**MOR**	0,9943	0,7841	0,7990	0,9997	0,9896
**POMC**	0,6123	0,1710	***0,0005	***<0,0001	***<0,0001
**POMC-MOR**	0,8916	0,1416	**0,0017	***0,0004	***<0,0001

Table T9: Significance values of PEAP test performed before PKC inhibitor injection (related to main Figure 6h).
Two-way ANOVA analysis followed by Dunnett's post-hoc test. 95%C.I. Source data are provided as a Source Data file.

Table T10

	GFP mice				
**Force**	**10**	**15**	**20**	**25**	**30**
**Naïve control**	0,3892	0,0525	0,5026	0,1829	0,1491
**MOR**	0,9328	0,9997	0,0722	0,9686	0,9997
**POMC**	0,5126	0,3034	0,3047	***<0,0001	***<0,0001
**POMC-MOR**	0,7534	0,2933	***<0,0001	***<0,0001	***<0,0001

Table T10: Significance values of PEAP test performed after PKC inhibitor injection (related to main Figure 6i).
Two-way ANOVA analysis followed by Dunnett's post-hoc test. 95%C.I. Source data are provided as a Source Data file.

Figure S12

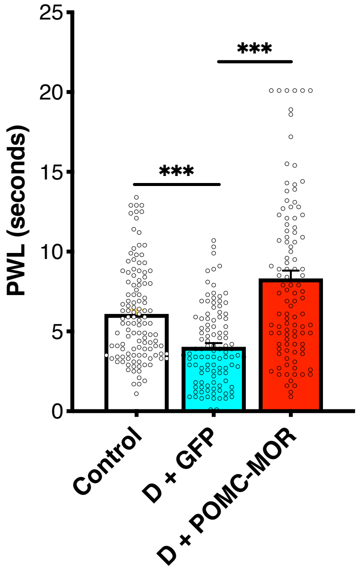

Fig. S12: Genetic reconstitution of POMC and MOR rescues thermal hyperalgesia in diabetic mice. (related to main Figure 7)

a Thermal hypersensitivity measured using Hargreaves test in naïve controls (non-diabetic, non-AAV injected) and diabetic mice overexpressing rAAV-9/2 constructs injected intrathecally. Readings after intraplantar injection of PKC inhibitor in both hindpaws (Gö6983, 20 µM, 45 minutes). Measurements were performed 6 weeks post STZ-induction in male mice (Naïve control, n=10; D+GFP, n=9; D+POMC-MOR, n=9, 4-5 readings per mouse). Data represents mean ± SEM; two-way ANOVA followed by Tukey's post-hoc test; ***p<0.0001) with 95%C.I. PWL=Paw Withdrawal Latency. Cyan bar: D+GFP, red bar: D+POMC-MOR. Circles represent individual data points. Source data are provided as a Source Data file.

Figure S13

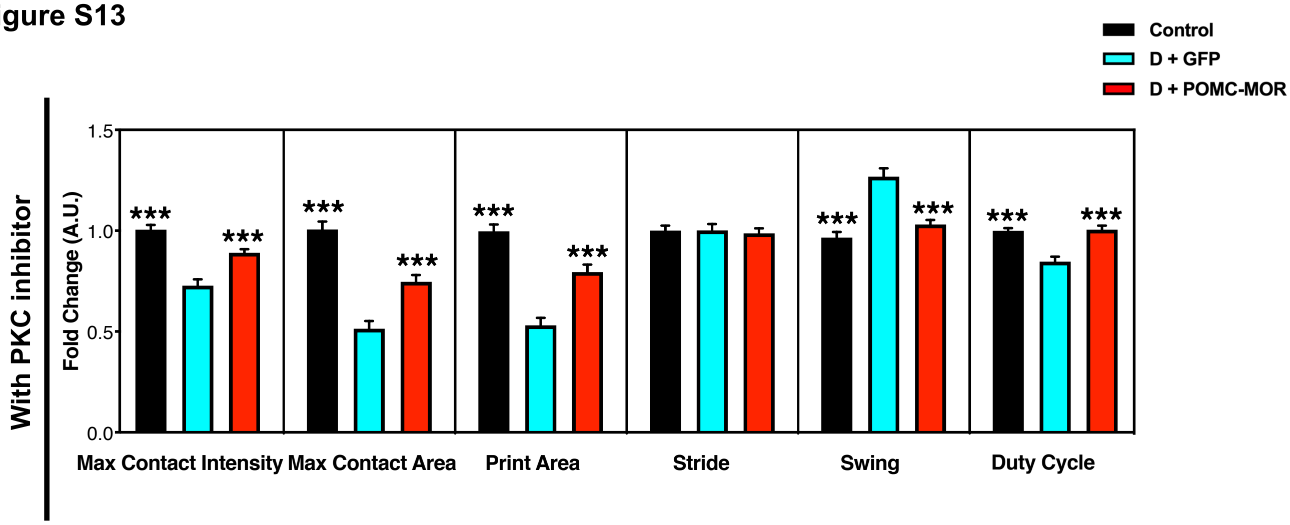

Fig. S13: Genetic reconstitution of POMC and MOR improves gait parameters of diabetic mice.
Gait parameters analyzed using Catwalk system in naïve control and male diabetic mice after intraplantar PKC inhibitor injection, (Gö6983, 20 µM, 45minutes). Measurements wer performed 6 weeks post STZ-induction in male mice. Gait parameters analyzed using Catwalk system in diabetic mice overexpressing AAV-9/2 constructs injected intrathecally. * represents significant difference compared to D+GFP cohort. (Naïve control, n=10; D-GFP, n=9; POMC-MOR, n=9; one-way ANOVA with Dunnett's post-hoc test). Data represents mean ± SEM; ***p<0.001 with 95% C.I. Cyan bar: D+GFP, red bar: D+POMC-MOR. Source data are provided as a Source Data file.

Table T11

Patient	Control	Type-1 Diabetic	Type-2 Diabetic
**Age (years)**	68.8 +/- 1.8	55 ± 20.9	71.6 +/- 2.7
**Gender**	male (n=5);<br>female (n=6)	male (n=4);<br>female (n=0)	male (n=6);<br>female (n=5)
**Diabetes**	no	yes	yes
**Reason for Amputation**	ischemia/Infection	ischemia/Infection	Ischemia/Infection
**Nephropathy/CKD**	in 27.3% cases	in 75.0% cases	in 72.7% cases
**Neuropathy**	absent	in 75.0% cases	present in 100% cases
**Retinopathy**	absent	in 50.0% cases	in 45.5% cases
**CVD**	in 54.6% cases	in 50.0% cases	in 72.7% cases
**Hypertension**	in 36.4% cases	present in 100% cases	in 54.6% cases

Table T11: Characteristics of patients analyzed for POMC and MOR expression in sciatic nerves (related to Main Figure 9).

Abbreviations: CRF= cardio-respiratory failure. CVD= cardio-vascular disease. CKD=Chronic Kidney Disease

Figure S14

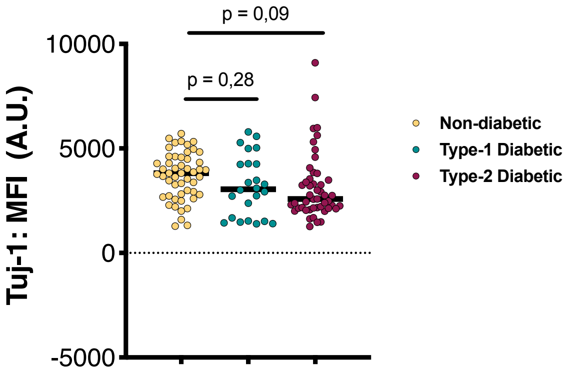

Fig. S14: Tuj-1 signal intensity is unchanged in the patient nerve sections of (related to Main Figure 9). Quantifications of anti-Tuj-1 signal intensity in sciatic nerve sections of non-diabetic healthy control, type-1 diabetic patients, and type-2 diabetic shows no change for the images quantified for POMC and MOR in the Main Figure 9 . (non-diabetic healthy control, n=11; type-1 diabetic patients, n=4 subjects and type-2 diabetic, n=11). Data represents dot plot with median; 3-7 sciatic nerve sections from each subject; one-way ANOVA followed by Dunnett's post-hoc test; with 95%C.I. Each circle represents individual data point. Orange circle: non-diabetic, teal circle: type-1 diabetic and maroon circle: type-2 diabetic patient data. Source data are provided as a Source Data file.

Table T12

Antigen	Species	Supplier	Application	Dilution
ß-actin	Rabbit	4967 (Cell signaling)	IB	1:4000
CD11b-Biotin	Rat	10120 (Biolegend)	IF (ms)	1:1000
CD45	Rat	103101 (Biolegend)	IF (ms)	1:1000
CGRP	Rabbit	24412 (Immunostar)	IF (ms and human)	1:4000 and 1:2000
FLAG	Mouse	8146S(Cell signaling)	IF (ms)	1:70
GFP	Mouse	11814460001 (Roche)	IF (ms)	1:200
Isolectin-B4	Biotin-conjugated	B1205 (Vector Labs)	IF (ms)	1:200
Lamp1	Rat	12601 (Biolegend)	IF (ms)	1:500
MOR (UMB3)	Rabbit	134054 (Abcam)	IP (ms)	
MOR	Rabbit	RA10104 (Neuromics)	IF (ms); IB	1:500; 1:1000
MOR	Rabbit	AOR-011 (Alomone)	IB (ms)	1:200
MOR	Guinea pig	NB100-1618 (Novus)	IF (human)	1:200
mCherry	Goat	AB0040-20 (Sicgen)	ICC	1: 500
NF-kB p65	Goat	Sc-109x (Santa Cruz)	ChIP; IB	1 µg; 1:500
NF-KB p65	Goat	100-4165 (Rockland)	EMSA	1 µg;
NF-kB p52	Rabbit	Sc-848x (Santa Cruz)	EMSA	1 µg
NF-kB p52	Rabbit	Ab175192 (Abcam)	ChIP; IB	1 µg; 1:500
NF-kB p50	Rabbit	Sc-114x (Santa Cruz)	EMSA	1 µg
NF-kB p50	Rabbit	Ab32360 (Abcam)	ChIP; IB; IF (ms)	1 µg; 1:2000; 1:1000
NF-kB c-Rel	Rabbit	Sc-70x (Santa Cruz)	EMSA; IB	1 µg; 1:1000
NF-kB c-Rel	Rabbit	PA5-47370 (Invitrogen)	ChIP	1 µg

Table T12: Primary antibodies
Abbreviations: IF= immunoflourescence, IB= immunoblotting, ICC= immunocytochemistry

Table T12 continued

NF-kB Rel B	Rabbit	Sc-226 (Santa Cruz)	ChIP	1 µg
NF-200	Rabbit	N4142(Sigma)	IF (ms and human)	1:300
Pan PKC	Rabbit	SAB4502356 (Sigma)	ICC	1:200
PGP9.5	Guinea pig	GP101014 (Neuromics)	IF (ms)	1:200
POMC	Goat	PA5 18368 (Thermo Fischer)	IF (ms, human); IB	1:100; 1:4000
POMC	Rabbit	23499 (Cell signaling)	IF(ms)	1:500
RNA pol	Mouse	8WG16/MMS-126R (Covance)	ChIP	1 µg
ßtubulin-III	Rabbit	T2200 (Sigma)	IF (ms)	1:500
ßtubulin-III	Mouse	14-4510-80(Invitrogen)	IF (human)	1:1000

Table T12: Primary antibodies
Abbreviations: IF= immunoflourescence, IB= immunoblotting, ICC= immunocytochemistry

SUPPLEMENTARY METHODS

Luciferase reporter assay

AtT-20 cells were trypsinized, washed and co-transfected with pGL3 constructs of either *WT* or *Mutant* promoter and Renilla luciferase construct as an internal control. 24 hours post transfection, medium was replaced with either 5 mM, or 20 mM glucose with reduced serum. 20 mM sorbitol exposure was included as an osmotic control. 6 hours post different glucose exposures, equal cell replicates transfected with both promoter constructs were then exposed to 10^{-8}M CRH and same number of replicates were kept unexposed for both constructs. 6 hours after CRH exposure (12 hours after different glucose treatments), reporter assay was carried using Promega dual luciferase assay kit according to manufacturer's instructions.

Kinase activity assay

To determine the activity of PKC kinase in the DRGs, protein lysates were prepared from freshly isolated DRGs (not frozen in liquid nitrogen) in RIPA buffer. The lysates were immediately used for the kinase activity determination. PKC kinase activity kit was used for this purpose (Enzo Lifesciences). The protocol described by manufacturers was followed.

Adeno-associated virus particle production

Recombinant AAV(1/2) particles were produced for overexpression of each of the following constructs: GFP (vector backbone), MOR-GFP, POMC-GFP and POMC-MOR-FLAG. The production of virions in HEK293 cells was performed as described previously [1]. In brief, HEK293T cells were seeded at 4 x 10^6 cells for each construct. Transfection mixture per construct was prepared as follows: tube, 5ml of 2x HeBs buffer (280 mM NaCl, 1.5mM Na_2HPO_4, 50 mM HEPES, pH 7.5), 500 uL of 2.5 M $CaCl_2$ and equimolar amount of pAM constructs and helper plasmids (pDP1rs and pDP2rs) to a final concentration of 37.5 µg. 2ml of the transfection mixture was added to each of the confluent 14cm culture dishes and incubated at 37°C for 24 hours, after which the media was removed and replaced with fresh complete DMEM.

96 hours post transfection, lysis buffer (50 mM Tris, 150 mM NaCl at pH 8.4) was added to the culture dishes and cell lysate was further subjected to free-thaw cycles between dry ice/ethanol bath and 37°C waterbath to release the virions. Lysate was treated with 50 units benzonase for 1 hour at 37°C and centrifuged to remove cell debris. The supernatant containing virus particles (=crude lysate) was passed through 0.45 μm syringe and concentrated using the Amicon-ULTRA filer (50 KDa cutoff).

Hotplate test

To measure thermal hyperalgesia of mice, each mouse was dropped on a hotplate at 50°C. A stopwatch was immediately started. The mouse was closely monitored for signs of distress, such as, licking of paw or lifting of paw. As soon as these signs were seen, the stopwatch was stopped and the mouse was lifted from the hotplate. The timings were recorded for each mouse thrice [2].

Gait analysis

Gait analysis was performed using Catwalk XT version 10.6. (Nodulus).

Definitions for each paw parameter analyzed:

- Paw print area: surface area of a complete print.
- Maximum contact intensity: maximum intensity of the paw at maximum contact. Intensity of the print depends upon contact between the paw and the glass plate. It increases with increasing contact. Therefore, this is a measure of the weight put on the glass plate.
- Maximum contact area: maximum area of a paw that comes into contact with the glass plate. In other words, it is the Print Area at maximum Contact.
- Swing: duration of no contact of the paw with the glass plate.
- Stride: distance between successive placements of the same paw.
- Duty cycle: is expressed as Stand as a percentage of Step Cycle

  Stand: duration of contact with the paw; Step cycle: Stand + Swing

REFERENCES

1. Njoo, C., Agarwal, N., Lutz, B. & Kuner, R. The Cannabinoid Receptor CB1 Interacts with the WAVE1 Complex and Plays a Role in Actin Dynamics and Structural Plasticity in Neurons. *PLoS Biol.* **13**, (2015).
2. Bannon, A. W. & Malmberg, A. B. Models of nociception: hot-plate, tail-flick, and formalin tests in rodents. *Curr. Protoc. Neurosci.* **Chapter 8**, Unit 8.9 (2007).
