# Supplementary material for: Loss of POMC-mediated antinociception contributes to painful diabetic neuropathy
Source: Nat Commun. 2021 Jan 18;12:426. doi: 10.1038/s41467-020-20677-0 (PMC7814083; doi:10.1038/s41467-020-20677-0)
Supplement: Supplementary file 7 — Description of Additional Supplementary Files [file 41467_2020_20677_MOESM7_ESM.docx]

Description of additional supplementary files

**Title: Supplementary Movie 1: Gait of control mouse**

Description: Video shows the gait of naïve control mouse analyzed using Catwalk system after intraplantar PKC inhibitor injection, (Gö6983, 20 μM, 45minutes). The speed in the video is double of the original speed.

**Title: Supplementary Movie 2: Gait of diabetic mouse**

Description: Video shows the gait of naïve diabetic mouse analyzed using Catwalk system after intraplantar PKC inhibitor injection, (Gö6983, 20 μM, 45minutes). The speed in the video is double of the original speed.

**Title: Supplementary Movie 3: Gait of diabetic mouse overexpressing GFP in ipsilateral DRG**

Description: Video shows the gait of diabetic mouse overexpressing GFP in ipsilateral DRG (right side) analyzed using Catwalk system after intraplantar PKC inhibitor injection, (Gö6983, 20 μM, 45minutes). The speed in the video is double of the original speed.

**Title: Supplementary Movie 4: Gait of diabetic mouse overexpressing POMC and MOR in ipsilateral DRG**

Description: Video shows the gait of diabetic mouse overexpressing POMC-MOR bicistronic contruct in ipsilateral DRG (right side) analyzed using Catwalk system after intraplantar PKC inhibitor injection, (Gö6983, 20 μM, 45minutes). The speed in the video is double of the original speed.
